# Evaluation of Complications of Heart Surgery in Children With Congenital Heart Disease at Dena Hospital of Shiraz

**DOI:** 10.5539/gjhs.v8n5p33

**Published:** 2015-08-20

**Authors:** Maryam Mirzaei, Samaneh Mirzaei, Elham Sepahvand, Afifeh Rahmanian Koshkaki, Marzieh Kargar Jahromi

**Affiliations:** 1 Faculty member, Jahrom University of Medical Sciences, Jahrom, Iran; 2 Booshehr University of Medical Sciences, Shahid Ganji hospital, Borazjan, Iran; 3 University of Social Welfare and Rehabilitation, Tehran, Iran; 4 Faculty of Nursing & Para-Medicine, Jahrom University of Medical Sciences, Jahrom, Iran

**Keywords:** congenital heart disease, surgery, complications, children

## Abstract

**Introduction:**

Today, with progress in the field of congenital heart surgery, different complicated actions are done in children. These actions may be associated with several complications, especially open heart surgery in which the cardiopulmonary bypass (CPB) is used. Serious complications can be caused high morbidity and mortality rates. Present study has been performed to determine the incidence of morbidity and mortality in cardiac surgery in children.

**Method:**

In a cross-sectional retrospective, records of 203 patients undergoing surgery for congenital heart disease in Dena hospital during 2013-2015 were reviewed for incidence of complications. Data was analyzed by using descriptive and analytical statistics and using SPSS version 18.

**Results:**

The mean age of samples was 3/65±4/47 years. The majority of samples (73/8%) were undergoing open surgery. The overall adverse cardiovascular complications were respectively, renal complications (44/3%), lung (40/3%), anemia (35/9%), heart (34/4%), gastrointestinal (17/2%), brain (14/2%), need for re-intubation of the trachea 11/3%), infection (7/8%) required reoperation (5/9%) and vascular complications (1/4%).

**Conclusion:**

High incidence of complications after congenital heart surgery makes necessary attention to complications and their treatment after surgery. It is necessary to apply the measures and careful monitoring of patients to minimize these effects.

## 1. Introduction

Heart disease is considered one of the most important threatening factors on the health of human society. The prevalence of heart disease in America is about 17 million patients per year. Of this amount, 64.7% include patients with coronary artery disease (CAD), 29.4% of the valve wastes, and about 5.8% have congenital heart malformations (Mangano, 1995). The prevalence of congenital heart defects is about 6 to 8 per thousand live births in which more than half of them require surgery during the first year of their life (Benson, 1989). The prevalence of congenital heart disease varies from country to country, for example, in America 6.61 per thousand live births, in the UK, 3.17, in Finland 1.95, in Denmark 6.18, in Sweden 3.57, in Australia 4.31 and in Canada 12.5 per 1000 is a live birth.

Unfortunately, due to the lack of recorded cases of congenital heart disease in Iran, no accurate figures on the prevalence and incidence of the disease exist (Zeinaloo, Tadbir, & TavakoL, 2002). In recent years, due to significant advances in cardiac surgery and postoperative care, the rate of mortality is reduced and age of surgery among children is decreased (Nouri, motaghi moghaddam, & Shah mohammadi, 2002). 45 types of congenital heart disease has been known so far, the most common are Ventricular Septal Defect (VSD) (25-20%), atrial septal defect (ASD) (13-8 percent), patent ductus arteriosus (PDA) (11-6%) and coarctation of the aorta (75%) are more prevalent (Behrmans & Kleigman, 2004). According to studies, in nearly one-third of the surgeries, at least one condition is detected (Benavidez, Gauvreau, Nido, Bacha, & Jenkins, 2007). Following these procedures, side effects such as cardiac complications, pulmonary, gastrointestinal, brain, blood, kidney, and so may be followed and even lead to death.

In open heart surgery, due to cardiomyopathy pulmonary bypass (CPB), which has different effects on different organs of the body, it is more likely to develop complications during or after surgery (Behrmans & Liegeman, 2004). Almost 400 thousand open heart surgery using pump cardiovascular (CPB) is done in the world which approximately 6% of these are children (Hill, Groom, & Bokhara, 1991). To better control of these complications and improve the prognosis of action, identification of mechanisms, incidence and risk factors play a major role.

Few studies on the complications of congenital heart disease surgery were performed in Iran. In a study conducted by Shah Mohammadi et al. (2001), the result of surgery on congenital heart defects in children admitted to Shahid Rajaie Hosipital for 20 years was investigated, but specific complications after surgery such as death toll are not taken into account and the complications such as mortality and epidemiological aspects have been considered (Shahmohammadi, Nouri, Ahmadi, & Molla Sadeghi, 2001).

Thus researchers decided to investigate the complications of surgery in children with congenital heart disease, according to the opening and closing actions in recent years and the complications are compared among them.

## 2. Materials and Methods

In a retrospective study of 203 patients with an age range of 02/0 (7 days) to 18 years from complications after heart surgery in congenital diseases were reviewed. The patients had undergone surgery in Dena Hospital at Shiraz during the years 2013 to 20015. Information about each patient were collected in a researcher made check list that consists of two parts. The data provided in the first phase include: age, sex, weight, diagnosis, type of surgery, length of hospital stay and duration of heart-lung bypass in open heart surgery. The second part of the check list contains a list of registered complications after surgery. Postoperative complications were divided into 10 groups. These effects include cardiovascular effects (decreased cardiac output, arrhythmias, atrioventricular block, the crisis of pulmonary hypertension, hypertension, and pericardial effusion requiring cardiopulmonary resuscitation (CPR), lung effects (atelectasis, pneumothorax, pleural effusion, chylothorax, hemothorax, diaphragm muscle paralysis, pulmonary hemorrhage, the need to Tracheostomy, aspiration, prolonged mechanical ventilation for more than 2 weeks), renal failure (acute renal failure, requiring dialysis, hematuria, blood urea and urinary tract infection), cerebral symptoms (seizures, loss of consciousness, stroke, abnormal movements, coma), gastrointestinal effects (GI bleeding, liver damage, and diarrhea), blood disorders (thrombocytopenia, increased PT and PTT, hemolysis), vascular complications (arterial or venous thrombosis), infectious complications, the need for repeated surgery and the need for endotracheal intubation. If the patient has at least one case of subgroups of each 10 main problems, he/she was considered as someone who has multiple effects. After collecting with the use of SPSS software version 11.5 and descriptive statistics (mean, standard deviation and range) and Statistics analytical (Chi-square and independent t test) the data were analyzed.

## 3. Results

In this study, records of 203 patients were studied. The mean age of the samples were 3.65 ± 4.47 years, about half of them (51.2%) were the boys. (Table 1-demographic information) who 150 patients (73.8%) underwent open heart surgery and 53 ones (26.3%) underwent closed heart surgery (Table 2, types of surgeries). The duration of cardiopulmonary bypass was 18 to 226 minutes with an average of 80.3 ± 46.2 minutes in open surgical procedures.

**Table 1:** Demographic characteristics of participants in the study

Variable	Unit	Frequency
Gender	Male	104(51.2%)
	Female	99(48.8%)
Age	Mean (SD)	3.65±4.47
Weight	Mean (SD)	12.97±10.84
Admission time	Mean (SD)	7.27±8.61
Operation type	Open	150(73.8%)
	Close	53(26.2%)

**Table 2:** Percentage and frequency of opening and closing procedures were reviewed in terms of postoperative complications

Types of surgical procedures		Number	Percent
	VSD^1^, ASD^2^, PS^3^, AS^4^, APW^5^	78	52
	AV chanal, TAPVR^6^	50	33.3
Open	ASO^7^, TCPC^8^, BGA^9^, Rastelli operatioin, Ross operation	22	14.7
sum		150	100
	PA banding, VSD PDA^10^	14	26.4
Close	PA banding, DORV VSD^11^	9	17
	Tetralogy of Fallot	15	28.3
	Pulmonary Atresia	15	28.3
sum		53	100

1 Ventricular Septal Defect

2 Atrial Septal defect

3 Pulmonary stenosis

4 Aortic Stenosis

5 Aorto pulmonary window

6 Total anomalous pulmonary Venous Return

7 Arterial Switch Operation

8 Total avo-pulmonary connection

9 Bidirectional Glenn Anastomosis

10 Patent Dactus Arterious

11 Double outlet Right ventricle

**Table 3:** Complications after heart surgery in children with congenital heart disease

Complications	Percent
Kidney	44.3
Lung	40.3
Blood	35.9
Cardiovascular	34.4
Gastrointestinal	17.2
Brain	14.2
The need for re- intubation of endotracheal	11.3
Infection	7.8

A total of 70 patients (43.4%) of the cases had complications after surgery. Complications of cardiac procedures include renal complications (44.3%), lung (40.3%), anemia (35.9%), heart (34.4%), stomach (17.2%), brain (14.2%), the need for re-intubation chip (11.3%), infection (7.8%) required reoperation (5.9%) and vascular complications (1.4%), respectively (Table 2). Among the kidney complications, the most common was urea hemoglobin (41.4%), and hematuria (27.1%), respectively. The most pulmonary of pneumonia complications (24.6%), the highest blood disorder was increased time of PT and PTT (1.28%), the most heart failure was reduced cardiac output (15.8%), atrioventricular node block (9.8%) and arrhythmias (7.4%). Among gastrointestinal side effects, the most common complication observed was liver damage (12.3%), followed by diarrhea had the highest complication (4.4%). Among the neurological complications, most complications observed were seizures and loss of consciousness (8.4%). 11.3% of the cases required re-intubation after surgery about 6% of the samples was re-operated. In 1.5% of the samples, vascular damage was observed and about 5% of them suffer from infectious complications with the clinical picture of sepsis.

## 4. Discussion

The study results showed that complications after surgery in children with congenital heart disease is the most common complication and the most ones observed was kidney complications. The most renal effect was blood urea (41.4%) and Hematuria (27.1%), respectively. Hemoglobin urea and Hematuria were commonly seen after bypass cardiopulmonary. It is due to hemolysis that blood urea often occurs during bypass. Long-term bypass, cardiotomy suction and obstruction excessive pump can lead to hemolysis resulting in increased levels of free hemoglobin. Existing hemoglobin in the urine is highly toxic to the kidney tubules and can form negatively charged proteins in the kidney tubules band and cast (Chang, Hanley, Vernowsky, & Wessel, 1990). The next complication was renal failure that was seen in 3.9% of patients. In many studies, including Hernick et al. (2011) (6%) and Rigden et al (1996) (5.3%), the incidence of kidney failure in children after heart surgery was less than 10 percent (Hornik, Jacobs, Li, Jaquess, & Jacobs, 2011. Rigden, Baratt, Dillon, Deleval, & Stark, 1982)

The second recorded complication that had the highest incidence followed by renal complications was pulmonary complications (40.3%). In the study conducted by Bailo et al. (2012), the highest recorded complication after surgery of congenital heart disease was pulmonary complications (23%) (Belliveau, Burton, O’Blenes, Warren, & Hancock Friesen, 2012).

In this study, the highest pulmonary complications observed were pneumonia (6.24%). However, in the study conducted by Hornick et al. (2011), the incidence of postoperative pneumonia was 2.2%, which is much less than what has been observed in the present study (Hornik, Jacobs, Li, Jaquess, & Jacobs, 2011). Also according to the studies, the incidence of postoperative pneumonia in children is relatively uncommon (Chang, Hanley, Vernowsky, & Wessel, 1990) and the incidence of nosocomial pneumonia (NP) in children after cardiac surgery varied between 9.6 to 21.5% (Healy, Hanna, & Zinman, 2011).

Patients at risk are those who have been Intubate before surgery or have a previous history of respiratory infections (Chang, Hanley, Vernowsky, & Wessel, 1990), in patients with a weakened immune system who have suffered cardio thoracic surgery are viral respiratory infections caused by lung infiltrations. Sean C. Shial respiratory virus (RSV) is considered as a major respiratory pathogen in children undergoing cardio thoracic surgery and can cause serious illness in children who have underlying lung disease or congenital heart disease (Avery, Long worth, 1995).

After pulmonary complications, blood disorders accounted for the next rank (35.9%), of which the biggest problem observed was increased PT and PTT time (28.1%). Increased PT and PTT time with an increased risk of postoperative bleeding in children is linked to several reasons for this issue.

One of the most important cause is the addition of heparin to blood flow during CPB to prevent clotting, coagulation disorders such as underlying disease, von-Willebrand, high thin blood, Immaturity of the liver, especially in infants and delays in the clearance of heparin and decreased levels of factor XII and XI, Prekallikrein and Kininogen wurth high molecular weight (Chang, Hanley, Vernowsky, & Wessel, 1990).

The next complication was heart problems (34.4%), of which decreased cardiac output (15.8%), atrioventricular node block (8.9%) and arrhythmias (7.4%) have assigned the most cases, respectively. In the study conducted by Shah Mohammadi et al. (1988), 39% of infants undergoing cardiac surgery, cardiac complications were observed that most of these effects were caused by arrhythmias and heart failure (Shahmohamadi, Nouri, Ahmadi, & Nikyar, 1998). In the study done by Hornick et al. (2011), arrhythmias has assigned 19.2%, reduced cardiac output 16.2% and block nodes AV 2.8% (Hornik, Jacobs, Li, Jaquess, & Jacobs,2011). Also, in the done by study Shanmogam et al. (2012), cardiac and arrhythmias effects assigned 14.5% higher risk of post-operative heart (Shanmugam, Clark L, Burton, Warren, & O’Blenes, 2012). Cardiothoracic surgery in children is often associated with decreased cardiac output in the postoperative period (Barker et al., 2013). Several reasons, including inadequate initial surgery, tamponade myocardial dysfunction, acute cardiac, increasing afterload and decrease cardiac output arrhythmias after heart surgery are discussed.

Whether in the form of Brady Arrhythmia or tachycardia arrhythmia, arrhythmias can decrease cardiac output. Mortality after cardiac surgery can be for both atrial and ventricular arrhythmias (Brian, Boyes, & Kirklin, 1993); so at least 48 hours after the surgery, the patient should be put under cardiac monitoring. The next complication was gastrointestinal side effects, of which the most complication observed was liver damage.

Postoperative liver damage often causes severe high blood pressure of preoperative right atrium, intraoperative hypoxia, and hypotension in the early stages after surgery and the amount of blood injected to the patient around the operation (Brian, Boyes, & Kirklin, 1993). The next complication was complications of brain that was mostly seizures and loss of consciousness (8.4%). In the early stages after heart surgery, the most common neurological disorders manifested is seizures and its incidence after cardiopulmonary bypass has been reported almost 4-15%. In many cases, the disorder is caused by damage created during surgery. In addition, an increased risk of cardiovascular - respiratory instability and also a major disturbance in cerebrovascular auto-regulation system occurred during this time may causes the patient prone to further neurological disorders (Bellinger, Jonas, Rappaport, & Wypij, 1995).

## 5. Conclusion

For the treatment of congenital heart diseases in children, surgery is the best and actually the final treatment option; but this method as other invasive procedures, is associated with some complications. Due to the high incidence of complications after surgery based on the results of this study, considering complications and their timely treatment are necessary actions after surgery that these complications can be reduced by applying necessary measures and accurate monitoring of the patients.
